# The role of microglia in human disease: therapeutic tool or target?

**DOI:** 10.1007/s00401-014-1330-y

**Published:** 2014-08-09

**Authors:** Nathalie Cartier, Coral-Ann Lewis, Regan Zhang, Fabio M. V. Rossi

**Affiliations:** 1 INSERM U986, 80 rue du Général Leclerc, 94276 Le Kremlin-Bicêtre, France; 2 MIRCen CEA Fontenay aux Roses, 92265 Fontenay-aux-Roses, France; 3 University Paris-Sud, 91400 Orsay, France; 4 The Biomedical Research Centre, University of British Columbia, Vancouver, BC V6T 1C7 Canada

## Abstract

Microglia have long been the focus of much attention due to their strong proliferative response (microgliosis) to essentially any kind of damage to the CNS. More recently, we reached the realization that these cells play specific roles in determining progression and outcomes of essentially all CNS disease. Thus, microglia has ceased to be viewed as an accessory to underlying pathologies and has now taken center stage as a therapeutic target. Here, we review how our understanding of microglia’s involvement in promoting or limiting the pathogenesis of diseases such as amyotrophic lateral sclerosis, Alzheimer’s disease, Huntington’s disease, multiple sclerosis, X-linked adrenoleukodystrophy (X-ALD) and lysosomal storage diseases (LSD) has changed over time. While strategies to suppress the deleterious and promote the virtuous functions of microglia will undoubtedly be forthcoming, replacement of these cells has already proven its usefulness in a clinical setting. Over the past few years, we have reached the realization that microglia have a developmental origin that is distinct from that of bone marrow-derived myelomonocytic cells. Nevertheless, microglia can be replaced, in specific situations, by the progeny of hematopoietic stem cells (HSCs), pointing to a strategy to engineer the CNS environment through the transplantation of modified HSCs. Thus, microglia replacement has been successfully exploited to deliver therapeutics to the CNS in human diseases such as X-ALD and LSD. With this outlook in mind, we will discuss the evidence existing so far for microglial involvement in the pathogenesis and the therapy of specific CNS disease.

## An introduction to microglia

Microglia constitute approximately 10 % of the total glial cell population within the CNS, with the density of these cells varying considerably between different anatomical regions, ranging from a high of 12 % in the basal ganglia to a low of 5 % in the cortex of mice [83]. Ramon y Cajal initially identified a population of cells distinct from neurons and macroglia (astrocytes); he designated “third element”, which was further divided into a main population representing oligodendrocytes, with the remainder of the cells defined as microglia by his student Pio Del Rio-Hortega (1919). Using silver carbonate staining of the embryonic brain, Rio-Hortega demonstrated that concentrations of mesodermal cells from the pia mater, which he referred to as “fountains of microglia” appeared on the surface of the fetal brain [40]. These cells had an ameboid morphology and at later stages of neurodevelopment dispersed throughout the brain rudiment, and differentiated to into cells with the stellate morphology characteristic of microglia.

Despite these early revelations regarding their ontogeny, for the better part of a century the identity of the cellular precursor of microglia remained an area of contention, with investigations into the origins of microglia generating three different hypotheses. In spite of Rio-Hortega’s early assertion that astrocytes and oligodendrocytes were of ectodermal origin, while microglia derived from mesodermal precursors, a growing body of evidence supported an alternative view that all glial cells derived from a common neuro-ectodermal stem cell progenitor, the glioblast [77]. In vitro studies reported the development of Mac1^+^ cells in primary astrocyte cultures created by disaggregating the murine neonatal cortex and clearing it of microglia by complement-mediated cell lysis, supporting the notion astrocytes and microglia originated from a common progenitor [108]. However, when astrocyte progenitors harvested from the rat subventricular zone were cultured under media conditions favoring microglia differentiation, mixed macroglial colonies consisting of astrocytes and oligodendrocytes were formed, none of which contained microglia [87].

Alternatively, it was postulated that microglia were of hematopoietic origin and were maintained through the recruitment of blood-borne monocytes. Evidence in support of their hematopoietic origin came in the form of irradiation/bone marrow (BM) transplantation studies in mice, in which genetically distinct BM donor cells were observed in CNS of recipient mice. However, later experiments demonstrated that in the absence of irradiation [100] and/or the intravenous injection of whole BM, which includes progenitor populations not found in the circulation under physiological conditions [3], BM-derived cell (BMDC) did not infiltrate the CNS. Thus, the accumulation of BMDCs in the CNS of chimeric mice was an artifact of the irradiation/transplantation paradigm used to create BM chimeras, and microglia are maintained through local self-renewal rather than through the recruitment of monocyte precursors from the blood. However, the developmental precursors that give rise to microglia were still yet to be identified.

A third hypothesis posited that microglia were the progeny of primitive hematopoietic cells originating in the yolk sac (YS) that colonized the brain rudiment during embryogenesis. Cuadros et al. [36] employed chimeras between chick embryos and quail yolk sacks (YS) to demonstrate that macrophages originating from the quail YS invaded that developing neuroectoderm of the chick embryo. A similar finding was reported by Alliot et al. [5] who showed microglia cells first appeared in the brain rudiment of mice at day E8.0. Based on this observation, the authors inferred that microglial progenitors must have originated in the YS, as this was the only site within the mouse embryo that contained cells with a macrophage phenotype before E8.0. However, at this point in time, the identification of microglial precursors had been muddied with observations made using irradiated BM chimeric mice and these studies were largely overlooked. It would be over a decade later and following the advent of fate-mapping techniques that the YS origin of microglia would be confirmed.

In their seminal work, Ginhoux et al. [51] utilized mice expressing a tamoxifen-inducible Cre recombinase under the control of a Runx1+ promoter and crossed them with the ROSA26-YFP Cre-reporter strain of mice. Runx1 is a transcription factor whose expression restricted to YS and embryonic liver hematopoietic progenitors. By treating pregnant females with tamoxifen at different stages of embryonic development, YFP would be expressed in a proportion (~30 %) of Runx1^+^ cells and their progeny, enabling the migration of YS cells to be mapped within the developing embryo. The authors found that when pregnant mice were treated with tamoxifen prior to E7.5, a time point at which Runx1^+^ cells are restricted to the YS, YFP^+^ macrophages were observed in the developing brain rudiment at E10, E13, and in developed CNS of 6-week-old mice in a proportion similar to that observed for YS macrophages. Notably, when pregnant mice were treated with tamoxifen at day E8.5, a time point at which definitive hematopoiesis within the embryo proper has mostly replaced YS hematopoiesis, Runx1 lineage traced cells were no longer observed in the CNS, indicating that microglia are the progeny of primitive macrophages from the YS that invade the developing CNS.

A limitation associated with the study by Ginhoux et al. related to the low efficiency (30 %) of Cre recombination was addressed in subsequent work by Schulz et al. in 2012 [135]. They utilized mice in which either the transcription factor Myb, required for the development of definitive hematopoiesis but not for myelopoiesis within the YS, or Sfpi1, required for development of macrophages, was deleted. They showed that the deletion of Myb did not affect the development of microglia, while the deletion of Sfpi1 resulted in the absence of all macrophages within the embryo, including microglia. Thus, in the absence of definitive hematopoiesis in the embryo, microglial development is not affected and primitive macrophages originating in the YS give rise to microglia. In 2013, Kierdorf et al. [76] identified the CD45^-^c-kit^+^ population of cells within the YS as noncommitted erythromyeloid progenitors of primitive YS macrophages and microglia.

Under resting conditions, microglia are dynamic cells that survey their surrounding microenvironment through the projection and retraction of their highly motile processes [110]. Detection of homeostatic imbalances, injury, or pathology within the CNS elicits a stereotypical pattern of activation. In their quiescent state, microglia express only low levels of CD45 and MHC proteins; however, upon activation the expression of these proteins is robustly upregulated [56], with a subpopulation of cells often observed to express CD11c [26, 55]. Along with these changes in surface phenotype, microglia undergo significant morphological changes, characterized by the retraction and thickening of their ramified processes and cell body hypertrophy [78]. Under conditions of neurodegeneration, populations of microglia acquire an ameboid morphology that is associated with increased phagocytosis, and serve to clear degenerative debris [58]. However, these surface phenotype and morphological changes are not an indication of the inflammatory gene repertoire expressed by these cells.

The CNS has several unique immunological features that together constitute immune specialization in comparison to other tissue compartments. The endothelium of the blood–brain barrier (BBB) strictly regulates the transmission of blood-borne substances and cells into the CNS, creating a sterile environment. Microglia exhibit a quiescent phenotype compared to other populations of tissue macrophages [92] and the CNS lacks an extensive lymphatic drainage system. The interplay between different cell types also contributes to maintaining this subdued immunological milieu. Neurons play an integral role in maintaining the quiescent state of microglia through the expression of membrane-bound ligands such as CD200 and CX3CL1, which function through cognate receptors expressed on the surface of microglia to down-regulate inflammatory gene expression [28, 63]. This immune specialization within the CNS serves to suppress and strictly regulate inflammatory responses that can damage surrounding neurons, which have only limited regenerative capabilities. Thus, in vitro studies of microglial activation must be interpreted with caution, as the phenotype of these cells is highly dependent on the specialized microenvironment within the CNS.

As will be discussed further, in the context of neurological diseases, microglia can promote neuronal survival or exacerbate the disease process depending on their activation profile, and are thus key players in influencing disease progression.

## Microglia in amyotrophic lateral sclerosis (ALS)

ALS is a fatal neurodegenerative disease characterized by the progressive loss of both upper (brain) and lower (brain stem and spinal cord) motoneurons, culminating in advancing paralysis. A variety of genetic mutations cause familial ALS, with a proportion (20 %) of these cases due to mutations in superoxide dismutase 1 (SOD1) [13]. Transgenic mice that over-express mutant SOD1 (mSOD) develop progressive motoneuron degeneration that is similar to ALS [57]. However, deleting the orthologous SOD1 in mice does not cause neurodegeneration, indicating that mSOD neurotoxicity operates through a gain of function mechanism [125, 136]. As with the vast majority of neurological diseases, neuropathology in ALS and the mSOD model is accompanied by robust microglial activation, and whether microglial activation has a correlative or causative role in neurodegeneration has been hotly debated [67].

Substantial experimental evidence has implicated microglial activation as a contributing factor to neurodegeneration in the mSOD model. In vitro studies have demonstrated that mSOD-expressing microglia stimulated with LPS produced greater amounts of pro-inflammatory factors including NO, superoxide, IL-6 and TNFα, and reduced levels of IGF-1 compared to similarly treated wild-type microglia [156, 161]. When microglia were co-cultured with motoneurons following activation by LPS, the increased production of NO and superoxide by mSOD microglia resulted in enhanced neurotoxicity compared to wild-type microglia [161]. However, removing microglia from the environment of the CNS significantly alters their behavior and due to the BBB, bacterial antigens such as LPS would not typically be encountered in the intact CNS.

To determine whether microglial dysfunction caused by mSOD expression contributes to causing neurodegeneration in vivo, Boillee et al. [24] utilized mice carrying a floxed-mSOD transgene crossed to strains expressing Cre recombinase to ablate mSOD expression in neurons or microglia. While investigators found that neuronal mSOD expression was required for the onset and early stages of disease, blocking mSOD expression in microglia significantly delayed the progression of disease and extended lifespan. Similarly, when the microglial compartment of mSOD mice was reconstituted with wild-type microglia, disease progression was slowed and survival was extended [16]. While investigators postulated these observations were due to the enhanced neurotoxicity of mSOD microglia compared to wild type, which had been demonstrated in vitro, an alternative explanation could be that mSOD expression in microglia reduces their neurotrophic potential.

Although these studies vilified microglia as drivers of neurodegeneration in the mSOD model, subsequent work casts doubt on their neurotoxicity. When proliferating CD11b+ microglia were selectively ablated in the CNS of mSOD mice, resulting in over a 50 % reduction in the number of activated microglia in the lumbar spinal cord, no effect was observed on disease progression [55], and gene expression profiling of spinal cord microglia from mSOD mice revealed that microglia maintain a primarily neuro-supportive phenotype throughout the course of disease [33].

Interestingly, the phenotype of microglia in mSOD mice evolved with disease progression, with cells exhibiting an neurotrophic phenotype at disease onset and a potentially neurotoxic phenotype by disease end-stage [89]. More recently, deep RNA sequencing of mSOD spinal cord microglia revealed that microglia co-express neurotrophic (e.g., IGF1, progranulin) and neurotoxic factors [34]. Thus microglia are primed to either delay or exacerbate neurodegeneration depending on the balance between the production of trophic versus toxic factors. Tipping this balance in favor of a neurotrophic phenotype may be a site for therapeutic intervention. Indeed, Neuraltus Pharmaceuticals is currently testing the efficacy of its drug NP001 in delaying the progression of ALS. Rather than universally dampening microglial responses, NP001 functions to skew microglial activation toward an M2 phenotype, enhancing the production of neurotrophic factors while down-regulating the production of neurotoxic substances. Phase 2 results demonstrated that in the high-dose patient group, there was no progression of disease in 27 % of patients treated, a 2.5-fold improvement over the placebo group (Neuraltus Pharmaceuticals Oct.29, 2012 release).

## Microglia in Alzheimer’s disease (AD)

AD is characterized by the degeneration of synapses and neurons, culminating in altered cognitive functions. The pathological hallmarks within the AD brain are senile plaques formed by the extracellular deposition and accumulation of beta amyloid (Aβ), and intraneuronal neurofibrillary tangles of hyperphosphorylated tau filaments [106].

The cause of neurodegeneration in AD has yet to be elucidated, though several pathogenic mechanisms have been postulated, including mitochondrial dysfunction and the accumulation of Aβ. Aβ is a peptide produced by the cleavage of amyloid precursor protein (APP), an integral membrane protein expressed by a multitude of cells but found in high concentrations at neuronal synapses, though the function of APP remains unknown.

As with many other neurodegenerative diseases, whether the inflammatory response associated with AD serves to drive or delay disease progression is a contentious and complex issue. Microglia are observed to cluster around Aβ deposits in AD brain and constitute a cellular component of senile plaques [97]. Notably, Aβ directly activates the classical complement pathway by interacting with C1q protein, triggering a complement cascade that results in the opsonization of plaques [129]. As microglia express scavenger receptors for opsonized targets, they are in turn activated and migrate toward senile plaques. Indeed, numerous scavenger receptors expressed by microglia have been shown to interact with Aβ including CD36 [75], class A1 scavenger receptors (Scara1) [74], and the receptor for advanced glycation end products (RAGE) [164], to name a few. Indeed, Frenkel et al. [50] demonstrated that ablating expression of *Scara1*, resulted in increased levels of Aβ deposition, while the pharmacological up-regulation of this receptor increased Aβ clearance.

In vitro studies showed that microglia stimulated with Aβ produced factors toxic to co-cultured neurons, while treating neuronal cultures with Aβ in the absence of microglia did not result in neuronal death [52]. While these results suggest that microglial activation in AD might serve to enhance neurotoxicity, later in vivo studies suggested otherwise. Overexpression of IL-1B in the brains of AD mice resulted in increased levels of microglial activation with a concomitant reduction of Aβ deposition in the hippocampus [138]. A protective function for microglial activation was further supported by the recent discovery that a variant in the gene encoding triggering receptor expressed on myeloid cells 2 (TREM2) increases the risk developing sporadic AD threefold [68]. TREM2 is expressed on the surface of myeloid cells, including dendritic cells, macrophages and is constitutively expressed by microglia. Loss-of-function mutations in the gene encoding TREM2 or the adaptor protein through which it signals (DAP12) results in Nasu-Hakola disease, characterized by bone cysts and progressive encephalopathy and neurodegeneration that culminates in cortical atrophy and early-onset dementia [109]. Takahashi et al. [152] demonstrated that ablating TREM2 expression in microglia inhibits their phagocytosis and elimination of apoptotic neurons and increases their production of TNFα and NOS2. Thus it has been hypothesized that in the context of AD, TREM2 genetic variants serve to reduce the ability of microglia to phagocytize Aβ and enhance their expression of pro-inflammatory factors, resulting in accelerated accumulation of Aβ and neurotoxicity [61].

Recently, microglial senescence has been postulated to play a role in the development of AD. With increasing age, the number of microglia in numerous areas of the murine CNS significantly increases, their cortical distribution becomes less organized and uneven [158], and microglial processes exhibit reduced branching and symmetry [153]. In the aged human brain, microglia possess an activated morphology [101], with some cells appearing dystrophic [149]. Functionally, aged microglia express increased levels of pro-inflammatory cytokines (e.g., TNFα, IL-1B) and reduced anti-inflammatory IL-10 expression [158]. Together, these changes suggest aged microglia are in a heightened state of basal of activation compared to their younger counterparts, suggesting that they would be more efficient at responding to and at removing accumulating Aβ. However, in vitro studies of microglia from the brains of aged AD mouse models demonstrated that despite the heightened activation of these cells, the expression of scavenger receptors that bind Aβ and Aβ-degrading enzymes, such as neprilysin and insulysin, is actually reduced [60]. Furthermore, an in vitro study comparing the ability of young and aged murine microglia demonstrated that aged cells take-up comparatively reduced levels of Aβ [111]. Thus a model has been suggested where microglia initially serve protective functions in the AD brain by phagocytizing Aβ; however, despite the increased accumulation of microglia with disease progression in brain, age-related microglia dysfunction reduces the clearance of Aβ, while the production of pro-inflammatory cytokines is increased [60]. Compounding this dysfunction is the observation that inflammatory cytokines (e.g., IFNγ, TNFα) serve to enhance the activity of both γ-secretase [90] and β-secretase, increasing the production of Aβ and the formation of senile plaques [163], and eventually creating a self-perpetuating cycle of inflammation and Aβ deposition.

## Microglia in Huntington disease

Huntington disease is caused by the expansion of a trinucleotide repeat within the Huntingtin (HTT) gene, leading to the formation of an extended polyglutamine stretch at the N-terminal of the protein Huntingtonin [130]. This leads to a progressive degenerative disease associated with loss of medium spiny GABAergic neurons in the striatum. While it has been widely thought that the mechanism underlying HD pathology is likely cell autonomous and dependent of neuronal expression of the protein, microglia activation takes place prior to the onset of overt symptoms, its accumulation in the brains of Huntington patients takes place early during disease progression [132] and correlates with disease severity [117]. This suggested that the involvement of the inflammatory system may be tightly linked to the underlying pathogenesis rather than simply being one of its outcomes [151]. Surprisingly, further analysis found that mutant HTT (mHTT) is capable of affecting the inflammatory system in a systemic fashion. An increase in inflammatory cytokines such as IL-6, IL-8 and TNFα was found first in the plasma of overt Huntington’s patients, and later in that of pre-symptomatic mutation carriers [37]. Further investigation in murine models carrying HTT transgenes including expanded repeats pointed to the fact that peripheral monocytes as well as alveolar macrophages, neither of which are thought to traffic through the CNS in homeostatic conditions [3], showed increased cytokine production in response to in vitro LPS stimulation [23].

A strong indication that cell autonomous effects are indeed at the basis of microglia hyper-excitability has been provided by the finding that expression of a mHTT transgene specifically in myelomonocytic cells is sufficient to induce such phenotype [35]. Surprisingly, however, and in contrast with previous literature, in this study, no effects of mHTT expression were seen on peripheral myelomonocytic cells. In addition, whether mice expressing mHTT specifically in microglia actually developed a disease mimicking Huntington was not reported, leaving the question of the pathogenic relevance of the observed changes open. Thus, while it is clear that microglia is altered in a cell autonomous way by expression of HTT containing expanded repeats, the relevance and mechanistic underpinning of such changes are not yet clear.

## Microglia in multiple sclerosis/experimental autoimmune encephalomyelitis

Multiple sclerosis is a chronic inflammatory disease with a significant autoimmune component that leads to demyelination and eventually progressive loss of functional neurons. Typically two phases of the disease can be recognized based on symptom progression: a relapsing–remitting phase in which damage is invariably associated with inflammatory infiltrates and a second “progressive” phase in which functional loss continues at a steady pace, sometimes without any signs of inflammation. Active lesions are always associated with the presence of activated macrophages and/or microglia [82], which has led to the widespread belief that these cells play an active role in MS pathogenesis. Whether such role entails causing damage or actually limiting it, or perhaps both at different stages of the disease, has been debated [67].

The most commonly used murine model of MS relies on the induction of autoimmunity by immunization of mice with specific myelin peptides and complete Freund’s adjuvant. There are several caveats with this model, and for the purposes of this review a main limitation is the fact that the nature of the inflammatory infiltrate observed in active lesions is significantly different from that observed in human samples. CD4T cells, rather than CD8, predominate and B cells are nearly absent. This last difference in particular is critical when considering the recent successes of specific anti-B cell therapies, which lead to a dramatic amelioration of MS-linked inflammation and can prevent acute relapses, even if they are eventually unable to arrest the disease progression [85]. Yet despite these caveats, it is from these experimental autoimmune encephalomyelitis (EAE) models that evidence for distinct roles of resident microglia and infiltrating cells arises.

One of the problems limiting our ability to gain a deeper understanding of the specific roles of microglia is the fact that despite a number of candidates having recently been proposed, we still lack a reliable marker to distinguish these cells from BM-derived macrophages. This distinction is particularly important in MS, as macrophages derived from infiltrating monocytes and microglia are both present in the CNS parenchyma [4].

Over the past few years, murine models have been developed that have led to significant progress. A model based on irradiation of parabiotic animals was developed in which circulating and resident cells can be easily distinguished. Using this model a temporal sequence was revealed in which massive microgliosis takes place before the development of severe symptoms and the concomitant infiltration of circulating cells. Indeed studies taking advantage of the fact that deleting the chemokine receptor CCR2 blocks the ability of monocytes to enter the CNS confirmed that their infiltration is an absolute requirement for the development of severe disease [4, 99].

**Fig. 1:**
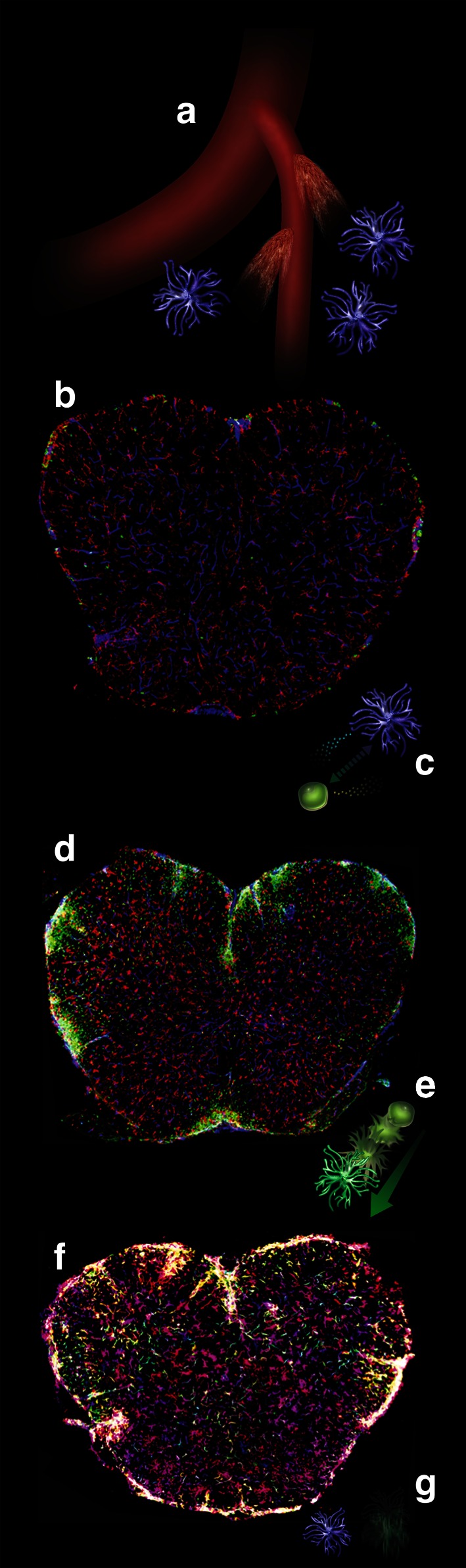
Illustration of the multiple roles of microglia during the initiation, progression and resolution of experimental autoimmune encephalitis. The spinal cord sections are derived from mice in which circulating cells and their progeny are marked by GFP expression (*green*), while tissue-resident microglia are not (see text). **a** Microscopic leaks in the CNS vasculature allow the extravasation of plasma components such as fibrinogen, which contributes to the induction of microglia expansion. **b** This leads to microgliosis well before circulating cells are capable of crossing the blood–brain barrier. Staining for the mature microglia/macrophage marker IBA-1 is shown in red, staining for CD31 (endothelium) in *blue*, circulating cells are green. **c** Expanded microglia play a role in attracting circulating inflammatory monocytes, possibly by the expression of chemokines such as CCL2/MCP1. **d** Inflammatory monocytes infiltrate the CNS parenchyma and, through a variety of mechanisms likely to involve myelin stripping and secretion of neurotoxic cytokines, exacerbate the damage and trigger progression toward severe disease. **e**, **f** Next, inflammatory monocytes mature into IBA-1 expressing macrophages, adopting a microglia-like morphology. This signals the beginning of remission. **g** Eventually, macrophages derived from circulating monocytes are completely cleared from the CNS, while microglia is reduced in numbers but persists to maintain the resident population of innate immune cells

The observation that microglial activation occurs early during disease suggested that while it may not be responsible for the majority of inflammatory damage, it might initiate a cascade of events eventually leading to the entry of deleterious inflammatory monocytes into the CNS. Another early event that has been linked to microglia activation is the disruption of the blood–brain barrier (Fig. 1a, b). The development of localized vascular permeability has been proposed to play a key role in microglia activation through the extravasation of serum components such as complement [124]. One of the most abundant serum components is fibrinogen. Beyond its well-studied role in coagulation, fibrinogen is also known to have powerful pro-inflammatory effects, partly by providing an adhesion substrate for CD11b, an integrin expressed on most myelomonocytic cells. Fibrinogen depletion has also been shown to reduce the severity of EAE, and this effect has been attributed to interference with its role in activating resident microglial cells [2]. More recently, fibrinogen extravasation has been linked to the formation of microglial perivascular clusters beginning in pre-symptomatic EAE and lasting through to peak of disease, and the presence of such clusters has been linked to disease progression [38]. However, fibrinogen depletion-mediated disruption of microglial clusters still allows EAE to proceed to mild symptomatic stages, which include microgliosis. This suggests that fibrinogen may have multiple roles, and that while it may not absolutely required for microglial activation it may influence monocytic infiltration and thus disease progression.

While the progeny of inflammatory monocytes is generally recognized as a key effector of demyelination in both autoimmune and other pathologies, a role for microglia in the pathogenesis of EAE has also been established. In a seminal paper in which microglia expansion had been prevented, mice failed to progress to severe disease upon EAE induction [59]. More recently, a revolutionary transgenic strategy allowing specific genetic manipulation of microglia and not of BM-derived cells has led to the clearest evidence so far that microglia are indeed involved in EAE pathogenesis. This strategy takes advantage of the fact that microglia self-renews in situ without involvement of BM cells, while short–lived circulating myelomonocytic cells are continuously replaced. Thus the lasting deletion of specific genes only in microglia can be obtained by transiently inducing CRE activity in myelomonocytic cells, and then waiting for the peripheral cells to be replaced by the progeny of undeleted stem cells. This strategy, which is significantly simpler than the parabiosis/irradiation approach mentioned above, allowed Goldmann et al. [54] to delete the signal transducer TAK1, involved in the activation of stress and of pro-inflammatory pathways through NF-kB, specifically from microglia. When these animals were subjected to EAE induction, they were found to be resistant to disease despite the fact that appropriate T cell responses were normally established in the periphery. In addition, the observation that cells lacking TAK1 lost the ability to activate CCL2 expression led the authors to hypothesize that one of the roles of microglia may be to attract inflammatory monocytes (Fig. 1c, d).

Another proposed role for microglia in EAE pathogenesis relates to antigen presentation. In MS, like in EAE, the repertoire of self-antigens against which T cells are capable of reacting increases during disease progression, and such “epitope spreading” is likely to play a significant role in exacerbating demyelination [84]. Epitope spreading has been shown to start from within the CNS rather than in peripheral lymphoid organs, suggesting that cells capable of presenting antigen and activating novel T cell clones must be present locally [98]. For efficient antigen-specific activation of T cells, an antigen-presenting cell (APC) expressing both antigen-loaded MHC and co-stimulatory molecules is required. While quiescent microglia do not express significant levels of these molecules at steady state, both MHC-I and MCH-II are upregulated in activated microglia, which also express co-stimulatory molecules such as CD40 and CD80/86 [71, 133]. When the ability of microglia to stimulate T cells was assessed in models of cuprizone-induced demyelination, it was found that they increased expression of MHC, the putative APC marker CD11c and co-stimulatory molecules as mentioned above, and that they were capable of efficiently presenting myelin peptides to T cell in vitro [126]. However, no evidence of T cell activation in vivo was found, pointing out limitations of assays that probe the ability of a given cell type to present antigen outside of its physiological context.

Finally, microglia have also been ascribed positive roles in EAE. These roles are mostly related to their ability to polarize to an alternatively activated, “M2” like state in response to type-2 cytokines, and to remove inhibitory myelin debris interfering with remyelination (Fig. 1e, f). Treatment of microglia with IL-4, a typical type-2 polarizing factor, reduces expression of pro-inflammatory genes such as TNFα and increases the expression of trophic factors capable of stimulating oligodendrocyte precursors such as IGFs. Delivery of IL-4 treated cells in the cerebrospinal fluid of mice led to a faster resolution of EAE [25]. In addition, microglia themselves express IL-4 upon activation, suggesting that it may play a role in inducing the M1 to M2 switch that the progeny of infiltrating monocytes undergoes just prior to EAE remission [4]. In support of a critical positive role of IL-4 in the CNS, mice lacking IL-4 or IL4Rα display exacerbated EAE [47], and this phenotype was not rescued by transplantation of wild-type BM in IL-4^−/−^ recipients, suggesting that resident radioresistant cells such as microglia are the critical source of this cytokine [120]. Interestingly, in this setting, both microglia and infiltrating cells lost expression of the M2 marker Ym1 supporting the notions that IL-4 may influence microglial fate in an autocrine fashion and that microglia-derived cytokines may modulate infiltrating cell fate.

## Microglia in X-ALD

X-linked adrenoleukodystrophy (X-ALD) is a metabolic genetic disease with a frequency of 1:17.000 males that causes progressive inflammatory demyelination in the brain. X-ALD results from mutations in the ABCD1 gene located in the chromosome Xq28. The ALD protein is a member of the adenosine triphosphate-binding cassette (ABC) transporter family [104, 105] expressed in the peroxisomal membrane. ALD protein loss of function results in the accumulation of unbranched saturated very-long-chain fatty acids (VLCFAs) in body fluids and tissues particularly brain and adrenal cortex [62, 66, 102]. Several distinct phenotypes occur frequently within the same family and are not correlated with ABCD1 gene mutations. The most prevalent form of X-ALD is a slowly progressive paraparesis with sphincter disturbances referred to as adrenomyeloneuropathy (AMN). AMN affects adult males after the age of 20 years and frequently heterozygous women after the age of 40 years. AMN is due to the involvement of the long tracts in the spinal cord leading to axonal degeneration. The cerebral form of X-ALD manifests in childhood as an acute cerebral demyelinating disease with neuroinflammation. The affected boys develop normally until 4–12, then cerebral demyelinating lesions start in the corpus callosum, internal capsules or brain stem and progress initially slowly over 1–3 years with mild neurocognitive deficits and no neurologic abnormalities. After this initial period, rapid cerebral demyelination occurs with a devastating clinical course. At this stage, brain MRI shows marked progression of demyelination and focal disruption of the blood–brain barrier. This rapid aggravation corresponds to the onset of inflammatory lesions, with infiltration and accumulation of macrophages and mononuclear cells behind the active edge of demyelinating lesions [44]. Most patients enter a vegetative state within 2–4 years of the first symptoms followed at varying intervals thereafter by death in 90 % of the cases. Whereas heterozygous women with AMN never develop the cerebral form (unless they have ABCD1 gene mutation on both alleles), 35 % of AMN males develop a cerebral demyelinating form of the disease between 20 and 35 years of age with the same outcome as in young boys toward vegetative state or death few years after the onset of neurologic symptoms.

The initiation of cerebral demyelination could be directly linked to the amount of VLCFA in complex lipids, such as phosphatidylcholines, sulfatides or gangliosides and to the inefficient degradation of VLCFA. This accumulation of VLCFA in myelin could lead to progressive destabilization of myelin sheaths and demyelination [62, 103]. Then this initial cerebral demyelination converts into a rapidly progressive inflammatory demyelination with invasion of macrophages containing myelin degradation products and opening of the blood–brain barrier [103].

Neuropathological analysis from ALD brains allowed major insight into the evaluation of the role of microglia in ALD [44]. Perivascular macrophages were shown to closely follow the leading edge of the demyelinating lesion. The region in which perivascular macrophages accumulate shows breakdown of the blood–brain barrier and accumulation of contrast material on gadolinium-enhanced magnetic resonance imaging (MRI). These blood-derived macrophages play a crucial role in the removal of myelin debris as revealed by the presence of recently degraded myelin lipids within them. Unexpectedly a zone lacking microglia within myelinated white matter was found beyond the leading edge. Given the spatial and temporal evolution of lesions in X-ALD, this loss of microglia could precede demyelination. Interestingly, the edge of the regions where microglia were absent showed activated microglia. This pattern of microglial activation is intriguing and offers clues to the pathogenesis of demyelination [44]. This area of activation is only seen in perilesional myelin, not in unaffected regions of the brain, and corresponds to areas where myelin contains higher levels of VLCFAs. Microglia in this region could be unable to degrade VLCFAs, and the cytotoxicity of lyso-phosphatidylcholine (LPC) containing VLCFA for macrophages and microglia could in turn lead to microglial activation and apoptosis [18, 44]. This microglial dysfunction and apoptosis might constitute an early pathogenic change in cerebral X-ALD, contributing to neuroinflammation and altering the neurovascular unit. Elevated levels of pro-inflammatory chemokines (CCL2, CCL4, IL-1ra, CXCL8) have been observed in the cerebrospinal fluid of patients with cerebral X-ALD and correlate with the MRI severity [93].

Altogether this suggests that, as observed in other neurodegenerative diseases like ALS, the process of demyelination in cerebral X-ALD might not be cell autonomous. Although the oligodendrocyte and the axon are the evident targets in cerebral X-ALD, the loss of microglia and/or abnormal microglia function could impair the ability to provide neuroprotective factors to deficient oligodendrocytes [53]. Injury to oligodendrocytes might be enhanced via an inflammatory response that follows tissue injury and plays an important role by initiating and accelerating the progression of the disease [53].

## Microglia dysfunction in Rett syndrome

Rett syndrome (RTT) is an X-linked CNS developmental disorder primarily affecting girls linked to loss-of-function mutations in the X-linked MECP2 gene encoding methyl-CpG-binding protein 2 [7, 31, 141]. RTT combines neuronal damage to non-cell-autonomous glial dysfunction. Neurons are primarily affected in the disease with dendritic and synaptic abnormalities [9, 112] and studies have initially supported an exclusively neuronal role for Mecp2 in RTT pathology. However, neuron-restricted expression of Mecp2 protein is insufficient to fully rescue the disease phenotype and glial cells were also shown to play a major role in the physiopathology of the disease [6, 12, 95]. Expression of Mecp2 in astrocytes was shown to arrest disease progression in Mecp2-null mice [91] and the toxicity of Mecp2-null microglia to neurons was suggested. Microglia was shown to release an abnormally high level of glutamate, causing excitotoxicity [94]. The role of microglia in RTT was addressed using BM transplantation Mecp2-null male mice. Surprisingly, pathology was halted and survival strongly increased (from 8 weeks to nearly 1 year) and animals showed remarkable improvement in several characteristic disease phenotypes including apnea, tremors, and locomotor performance. Myeloablative conditioning of the brain itself had no beneficial effect. Since major impairment in phagocytic ability of primary Mecp2-null microglia was evidenced in vitro, it was suggested that insufficient clearance of debris within the brains of these animals could contribute to the severity of the pathology and that phagocytic clearance by microglia could be important to improve the disease [41, 42]. Microglia could induce neuronal injury then subsequently fail to remove properly neuronal debris. This impaired phagocytic activity of microglia may not only contribute to RTT, but more largely to the pathophysiology of other CNS disorders. This is suggested by the observation that TREM2 myeloid cell receptor dysfunction is implicated in Nasu-Hakola disease, a pathological condition that shares similar phenotypic abnormalities with RTT [116]. TREM2 signaling was shown to facilitate debris clearance by microglia [152]. These data have strong implications for the treatment of RTT. A more detailed description of the role of microglia in RTT is available in this issue.

## Microglia replacement as a therapeutic tool for CNS diseases

### Turn over of brain microglia after hematopoietic cell transplantation (HCT)

Substantial debate remains on the existence, the modalities, and the kinetics of CNS microglia replacement by hematopoietic-derived cells after HCT [3, 100]. Many in vivo studies suggest that microglia are maintained after birth by local precursors that colonize the brain before birth independently from circulating mononuclear cells [3, 51, 100, 122, 142]. However, studies in irradiated mice demonstrate that an increased microglial engraftment of BM cells is observed after brain lesions suggesting that recruitment of BM-derived microglia (BMDM) occurs in many types of neuroinflammatory, neuroinfectious or neurodegenerative processes with or without alteration of the blood–brain barrier [39]. Thus neuroinflammation and microglia activation could promote recruitment of BM-derived cells to the CNS [119, 131]. Multiple examples comprise not only stroke [122] neurodegenerative diseases like Alzheimer’s disease [143], ALS [16, 70, 88, 146], Parkinson’s disease [128], multiple sclerosis [49] leukodystrophies [159] but also focal neuronal degeneration, traumatic brain injury [144] or experimental neuropathic pain. These BMDM are clearly distinct from CNS-infiltrating terminally differentiated macrophages [16, 122, 143, 157].

Experiments performed in myeloablated mice reconstituted with green fluorescent protein (GFP+) BM cells showed that both perivascular macrophages and microglia were replaced, to some extent, by BM-derived cells [10, 43, 122, 142, 159]. These studies showed progressive reconstitution of a significant fraction of differentiated, resting microglia a few months after HCT. However, the turn over of brain microglia after HCT is a slow process. Indeed, 3 months after reconstitution, the engraftment rate of Iba1 positive donor-derived microglia was estimated to reach 25 % 12 months after transplantation [30, 121].

### Microglia depletion as a tool to create a monocyte/microglia niche in the CNS

Busulfan conditioning regimen was recently shown to allow efficient turnover of microglia with donor-derived elements by modulating the local brain environment, likely by creating “space” for the engraftment through depletion of endogenous microglia [19, 88]. These experiments mimic the concept of myeloablation used in HCT procedures. The identification of donor-derived cells proliferating in the brain long-term after HCT and the appearance of progressively more mature donor cells [10], suggest that following myeloablation (albeit not in physiologic conditions) myelomonocytic reconstitution of the CNS can result from expansion and maturation of donor-derived precursor cells that are able to migrate in the brain short-term after HCT and proliferate locally. This is an important finding that could explain the efficacy of hematopoietic stem cell (HSC) gene therapy observed in X-ALD. In this trial, comparable efficacy of gene therapy to that described in ALD subjects showing full donor chimerism after allogeneic HCT was observed despite a limited engraftment in the BM and a number of corrected circulating mononuclear cells below 15 % [30]. HSCs might be required for obtaining microglia replacement by the donor because they comprise a cell subpopulation able to replace the intraparenchymal microglia precursors that are eliminated upon myeloablative conditioning, rather than because they ensure long-term hematopoietic donor engraftment in the BM [3, 19].

### Using monocytes to potentially replace dysfunctional microglia

Supporting the view that mature monocytes are a potential source of BMDM, several in vitro studies demonstrated that murine or human mature monocytes differentiate into microglia-like cells when co-cultured with astrocytes [86, 134] and BM-derived immature monocytic cells commit toward microglia-like cells under similar experimental conditions [137]. These microglia-like cells harbor several features of native microglia such as a ramified morphology, the presence of microspikes and filament-like structures detectable by electron microcopy, as well as a characteristic pattern of ion channels [73, 134].

By inducing microglia depletion in vivo, rapid repopulation of the brain with new Iba-1-positive cells was obtained within 2 week [155]. The engrafted cells expressed high levels of CD45 and CCR2 and pattern of expression suggesting they consisted of peripheral monocytes. Although two times more numerous, the overall distribution of the engrafted cells was remarkably similar to that of microglia. These findings suggest that blood-derived monocytes infiltrate and engraft in the microglia-depleted mouse brain regions, having the potential to occupy the adult CNS myeloid niche normally inhabited by microglia [155]. Myeloid cell engraftment was rapid and appeared limited to the period of microglial depletion, but the presence of newly engrafted cells remained remarkably stable over time (27 weeks) suggesting these cells are long-lived. These cells were capable of migrating to and sending processes toward acute lesion of neuronal damage, in similar fashion as microglia [8, 167] and phenotypic characterization revealed similarities with resident microglia. These findings suggest that, in microglia-depleted adult brain the newly engrafted myeloid cells could perform surveillance and scavenging functions similar to microglia.

These cells could exert therapeutic effects particularly in specific circumstances when selective loss and dysfunction of microglia or microglia senescence are observed in disease processes and in the aged brain. Since resident microglia appear to degenerate during aging-related disorders such as Alzheimer’s disease [48, 148], one might consider the use of BM myeloid cells or monocytes to compensate the defective functions of senescent resident microglia. Indeed, in a model of Alzheimer’s disease BMDM were shown to be more efficient than resident microglia in eliminating amyloid plaques [143, 145]. A beneficial effect of monocytic engraftment in CNS tissue was also demonstrated in spinal cord injury [140].

### Microglia enzyme factory to treat Lysosomal storage diseases

Lysosomal storage disorders (LSDs) comprise a heterogeneous group of more than 40 inborn errors of metabolism caused by mutations in genes encoding soluble lysosomal hydrolases, integral membrane proteins and transporters and characterized by tissue substrate deposits [1, 107]. Protein deficiency results in abnormal catabolism of specific byproducts and impaired intracellular turnover of a broad range of molecules including sphingolipids, glycoproteins glycosaminoglycans, and glycogen, leading to the accumulation of these substrates in tissues and organs. This substrate accumulation in lysosomes affects the architecture and function of the cells and the tissues. Most lysosomal enzymes are ubiquitously expressed and their absence causes a broad spectrum of systemic and CNS manifestations. CNS involvement is observed in up to 70 % of LSD, leading to neurodegeneration and/or dysmyelination. The severity of the CNS disease ranges from mild mental retardation with minimal alteration of cognitive function to severe and rapidly progressing disability and death.

Interestingly, in several LSDs the primary protein defect may be further exacerbated in the CNS by microglia activation that develops as a primary reaction to substrate accumulation within these cells and/or an inflammatory response to a primary neuronal damage caused by the disease [150].

Most of therapeutic approaches developed for LSD take advantage of the trafficking of lysosomal enzymes and a phenomenon referred to as “cross-correction”. Correct trafficking of lysosomal enzymes requires posttranslational modifications [113]. Newly synthesized enzymes are glycosylated in the endoplasmic reticulum and then phosphorylated in the Golgi apparatus on the 6 position of the terminal mannose residues (M6P) and sorted as inactive proenzymes to the lysosomes. A fraction escapes this lysosomal sorting pathway and is secreted in the extracellular space [1]. The secreted proenzyme can be recaptured by neighboring or producer cells using the M6P receptor expressed on the cellular membrane. Current therapeutic approaches take advantage of this secretion/uptake system to provide an exogenous supply of the missing enzyme that can be taken up by target cells and thus correct the metabolic defect.

A major therapeutic advance for the treatment of LSD has been the parenteral injection of recombinant proenzyme, a strategy called enzyme replacement therapy for those LSD with no involvement of the CNS, like non-neuropathic Gaucher, Fabry, or Pompe diseases [14, 15, 45, 69]. However, this strategy is non-efficient to correct CNS involvement, since the blood–brain barrier limits the access of the systemically delivered enzyme to the CNS.

### Replacing microglia using HCT to treat LSD

A significant advantage of HCT is that donor-derived, enzyme-producing cells are able to migrate to the brain. HCT for LSD has been performed for nearly 30 years, with the goal to repopulate recipient hematopoietic system with cells expressing the functional missing enzyme and stop the slow evolution of the disease and prevent the onset of clinical symptoms [10, 46, 118, 119, 147]. In that strategy, replacing deficient macrophages and microglia by normal cells combines the advantage of furnishing the tissues and particularly the brain with a critical enzyme source, with the potential restoration of scavenger function [43, 64, 81].

The first proof of efficacy of this strategy to stop the evolution of LSD was made in Hurler syndrome (MPS I). Mucopolysaccharidosis (MPS) are a family of inherited disorders caused by deficiency of lysosomal enzymes needed to degrade glycosaminoglycans (GAGs). Since the first transplantations performed 30 years ago [107], major beneficial results have been obtained in patients with Hurler syndrome [79, 147, 150]. From these impressive results, it was expected that all MPS could be treated by HCT. However, benefit from HCT only appears to be significant for MPS VI (Maroteau-Lamy) [80] and VII (Sly) [162] although the reasons for this remain unclear. Efficacy of HCT differs between the different storage diseases, in part because the recruitment of macrophage/microglia, mediated by chemokines that facilitate the migration of blood-derived cells to the brain differs in the different pathologies [160]. Beside MPS, HCT has beneficial effects in pre-symptomatic or late-onset Krabbe disease, and in the attenuated forms of metachromatic leukodystrophy [69]. Overall, the therapeutic effects of HCT in LSD with CNS involvement depends on the severity and on the time course of the disease [15]. The best results are obtained when HCT is performed at a very early stage of disease progression, or even in pre-symptomatic patients early identified because of family history. Efficacy is strongly dependent on the delay between the onset of the symptoms and the transplantation procedure, and limits the indications of allogeneic HCT [20, 22]. This underlines the need for defining optimal conditioning to improve microglia renewal from donor cells early after transplantation (see above) [27]. Microglia is a site of storage and a critical player in disease neuropathology, and could be particularly sensitive to the toxic effect of busulfan, as suggested by the higher donor chimerism observed in LSD models after HCT [21, 131].

### Hematopoietic cell gene therapy to overcome the limits of HSC efficacy in LSD

**Fig. 2:**
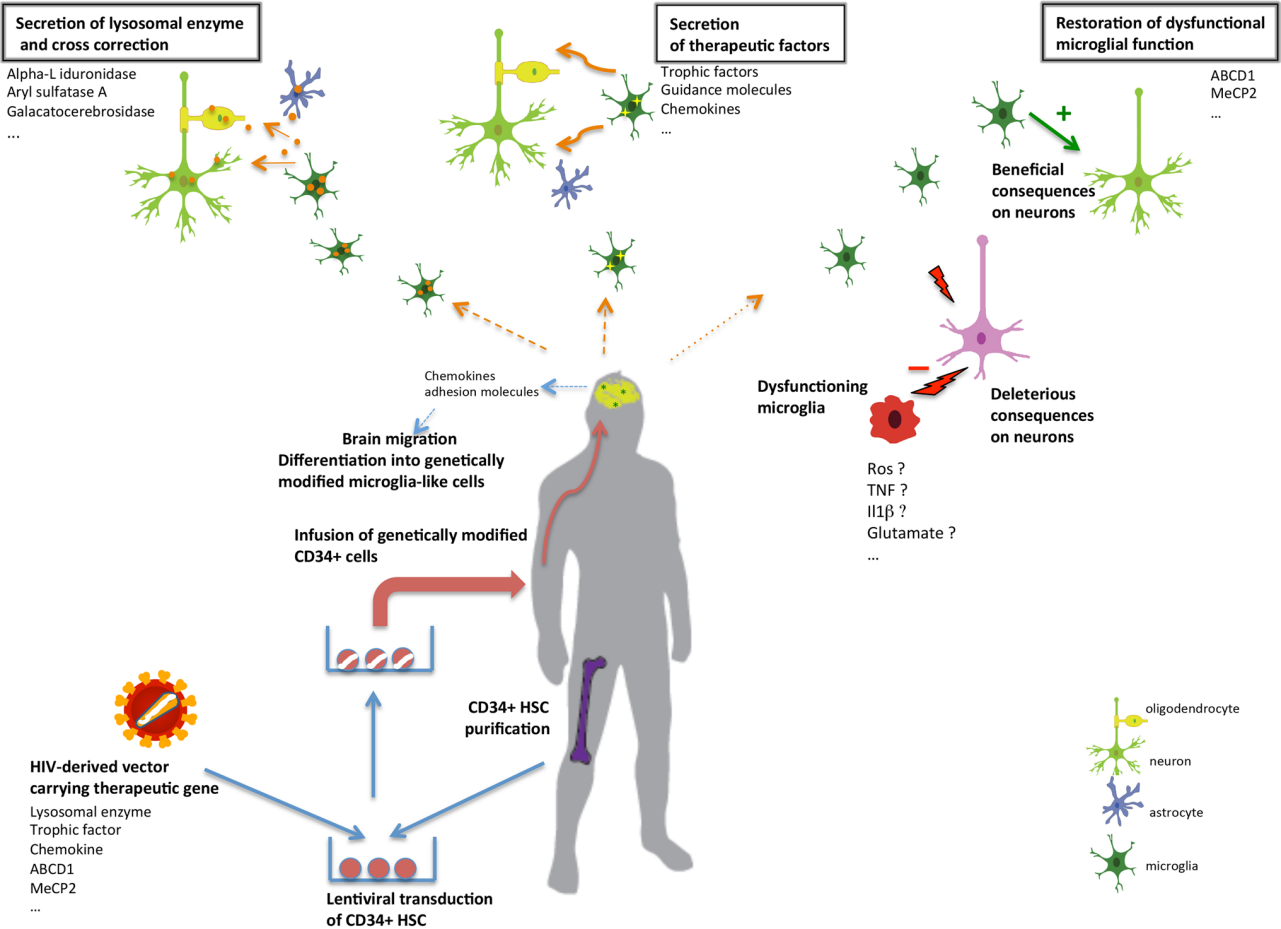
Transplantation of genetically modified hematopoietic stem cells to treat CNS diseases. After harvesting hematopoietic cells from the bone marrow of the patient, the CD34^+^ cell fraction containing hematopoietic stem cells (HSC) is purified and transduced with a lentiviral vector carrying the therapeutic gene. This gene can be the normal version of a deficient gene in patients with genetic diseases (ABDC1 in X-ALD, MeCP2 in Rett syndrome, lysosomal enzymes) or genes coding proteins of therapeutic interest like trophic factors, chemokines, and guidance molecules. Transduction with the lentiviral vector leads to stable integration of the therapeutic gene in the population of stem/progenitor cells. Genetically modified cells are re-infused into the patient and reconstitute the bone marrow stem cell compartment. A fraction of stem cells or myeloid progenitors is able to migrate to the CNS, cross the brain barrier and locally differentiate into genetically modified microglia-like cells. Chemokines (cell migration/chemotaxis inducing cytokines), such as monocyte chemoattractant protein 1 CCL2/MCP-1 and fractalkine CX3CL1/Fkn, produced by neurons, astrocytes and microglia, appear to attract peripheral blood mononuclear cells (PBMC) across the BBB into the brain parenchyma. Particularly, monocyte chemoattractant protein 1 (MCP-1) is and its receptor CCR2 have been implicated in a number of inflammatory diseases and are likely to be essential in this recruitment of bone marrow-derived cells. Migration of blood peripheral blood monocytic cells (PBMC) across the BBB into the brain parenchyma also depends on increased expression of various adhesion molecules, such as VCAM-1 ICAM-1, IG9 and E-selectin, all of which may promote PBMC attachment and transmigration. In the case of lysosomal diseases, lentivirus-mediated lysosomal enzyme overexpression allows efficient cross-correction of neurons and glial cells with diffusion of the therapeutic effect (*left*). HSC can also be engineered to express trophic factors with potential beneficial effects on surrounding neurons or glial cells (*center*). In diseases like X-ALD or Rett syndrome, the correction of deficient microglial function by transplantation of cells genetically modified with lentiviral vectors allows mitigation of neuronal damages induced by endogenous dysfunctioning microglia (*right*)

Besides safety (reduced risks associated with allogeneic transplantation and particularly graft versus host disease), the major advantage of the use of genetically corrected autologous compared to allogeneic HCT is the possible improvement of the therapeutic potential due to higher expression of the missing enzyme compared to the normal level (Fig. 2). Preclinical evidence of efficacy was obtained in MLD and GLD (globoid leukodystrophy) for which allogeneic transplant was shown to be inefficient [115, 122]. Based on these results a clinical application was developed in patients with early forms of MLD leading to very encouraging results with enzyme activity restoration at above-normal levels in the cerebrospinal fluid and substantial clinical benefit in treated patients [13].

### HCT to replace deficient microglia in X-ALD

Long-term follow-up have confirmed that hematopoietic cell transplantation (HCT) can arrest the neuroinflammatory demyelinating process of X-ALD when the procedure is performed at an early stage of the disease (minimal neurologic and neuropsychological deficits, limited extension of demyelinating lesions at brain MRI [11, 96]. However, demyelinating lesions continue to expand after transplantation usually for 12–18 months, before this progression is arrested. This delay in the benefit of HCT is likely due to the slow replacement of brain microglia from BM-derived cells [30, 72]. Experiments in vivo in X-ALD mouse showed that transplantation of Sca-1 hematopoietic stem cells resulted in the replacement of only 20–25 % of brain microglial cells 12 months after transplantation [30]. Inflammatory demyelination and microglial cell death allow long-term repopulation of the brain parenchyma with macrophages/microglia derived from peripheral hematopoietic cell progenitors [27, 165, 166]. The integrity of the blood–brain barrier is probably an important issue for HCT efficiency in ALD, since most successfully transplanted patients had enhanced contrast of cerebral demyelinating lesions after intravenous injection of gadolinium reflecting abnormal blood–brain barrier. This confirms mice data suggesting the need of blood–brain barrier disruption for myeloid progenitors to penetrate into the brain and differentiate into microglia-like cells [3, 29, 99, 100].

The mechanism by which allogeneic HCT arrests the cerebral demyelinating process and brain inflammation in cerebral X-ALD is still incompletely understood. The conditioning regimen has no effect by itself [139]. From the series of 36 ALD patients that received allogeneic HCT in France, four patients with no or delayed engraftment after full myeloablating conditioning regimen with busulfan and cyclophosphamide uniformly suffered devastating progression of cerebral demyelination [29]. In contrast to HCT performed for LSD, there is no possible cross-correction of other cell types after HCT in X-ALD since ABCD1 peroxisomal membrane protein cannot be secreted. This is confirmed by HCT experiments in X-ALD mouse. The murine model of X-ALD accumulates VLCFAs in all tissues, including the brain [123]. HCT in ALD mouse does not correct the accumulation of VLCFA in the brain, confirming the absence of metabolic cross-correction, in contrast to CNS lysosomal diseases. However HCT corrects oxidative stress damage in the spinal cord (N Cartier and A Pujol, unpublished results). Allogeneic transplantation in X-ALD probably corrects the abnormal function of brain microglia, even it is still unknown if this deficiency is directly related to the accumulation of VLCFA.

### Hematopoietic cell gene therapy in ALD

Allogeneic HCT remained associated with significant risks of severe graft vs. host disease and prolonged immune deficiency and a high mortality risk [32, 65]. Transplantation of autologous HSCs genetically modified to express the missing ALD protein may circumvent these severe complications (Fig. 2). In vitro experiments of ALD gene transfer with lentiviral vectors have shown biochemical correction of monocytes/macrophages derived from transduced ALD-deficient human CD34+ cells [17]. In vivo, the xenotransplantation of lentivirally transduced human ALD CD34+ cells into non-obese diabetic/severe combined immunodeficient (NOD/SCID) mice demonstrated that these human genetically modified hematopoietic cells were able to migrate into the brain of transplanted mice and to differentiate into microglia-like cells expressing the human ALD protein [10, 17]. Four X-ALD patients who were candidate for allogeneic HCT but had no HLA-matched donor have been treated using HSC gene therapy with lentiviral vector [30]. The number of hematopoietic cells expressing ALD protein reached around 25 % of peripheral blood cells from the treated patients 30 days after infusion of the transduced CD34+ cells. This percentage decreased with time but stabilized between 12 and 16 months to 10–15 %. Despite this limited number of corrected hematopoietic cells, the results of hematopoietic cell gene therapy in X-ALD with up to 7 years follow-up are comparable to the results of uncomplicated allogeneic transplantation in terms of disease outcome, with no side effects particularly no genotoxicity [30] (P Aubourg and N Cartier, unpublished results). These beneficial results could, in part, be explained by the fact that the ALD protein was overexpressed in HSCs and myeloid progenitors capable of colonizing the CNS. Although there is no evidence of a selective growth advantage of corrected microglia-like cells in ALD, their precursors might have some advantage in expanding within the ALD brain environment. As previously discussed, it is also possible that brain microglia cells from treated patients might be replaced by infused short-lived progenitors that contain a higher proportion of gene-corrected cells.

The fact that only partial correction of the hematopoietic cells is sufficient to stop the progression of the cerebral disease is of importance for future therapeutic indications. The mortality risk of HCT performed with HLA-matched unrelated donor or cord blood remains close to 15–20 % in children and 30 % in adults when using full myeloablative conditioning regimen commonly used in ALD with cyclophosphamide and busulfan. Reduced intensity conditioning (RIC) regimen with a lower risk of toxicity could possibly allow to propose HCT to ALD patients at a slightly more advanced stage of cerebral demyelination and in adults in whom a full myeloablation is associated with significant toxicity and mortality risks [29, 114, 127, 154]. However, this should be balanced with recent results in mice suggesting that the use of busulfan-based conditioning could favor a sustained brain chimerism of the transduced cells, potentially higher than the one detected in the hematopoietic system [27].

## Conclusion

The fact that microglia play multiple roles in human disease is well established. Defects in microglia can lead to their inability to perform some physiological functions such as debris clearance or enhance other functions, such as the secretion of neurotoxic factors, to the point in which they contribute to the establishment of pathogenesis. Thus in a large variety of pathologies microglia are thought to be promising targets for therapeutic intervention. Yet, due to our growing but still incomplete knowledge of the mechanisms underlying their workings, our success in identifying strategies that modulate microglial function in vivo has been limited. The most successful therapeutic approaches so far have been those that exploit the ability of BM-derived myelomonocytic cells to colonize microglial niches within the CNS in a myeloablation/transplantation setting. These cells can be engineered to produce disease-modifying factors, such as specific enzymes missing in lysosomal storage diseases, thus serving as long-term drug delivery factories for the treatment of neurological diseases, or restore microglia dysfunction, thus serving as real cell therapy. In all these applications, microglia replacement can play a critical role in many CNS diseases that currently lack efficacious treatments.
